# Supplementary material for: A Modular Workflow for Model Building, Analysis, and Parameter Estimation in Systems Biology and Neuroscience
Source: Neuroinformatics. 2021 Oct 28;20(1):241–59. doi: 10.1007/s12021-021-09546-3 (PMC9537196; doi:10.1007/s12021-021-09546-3)
Supplement: Supplementary file 1 — Supplementary file1 (DOCX 519 KB) [file 12021_2021_9546_MOESM1_ESM.docx]

## Supplementary Materials

We tested the Subcellular Workflow by running additional models of varying degree of complexity through our tools and various simulation environments discussed in the main manuscript. We imported a pre-existing model of the MAPK cascade (reactions from the green box from [Fig. 7A] from the FindSim workflow in Viswan et al. 2018)^^[[1]](#footnote-1)^^. The model has 102 species, 102 reactions and 160 parameters. We rebuilt the model in SBtab and reproduced simulations of the two test experiments from Viswan et al. 2018 [Fig. 7B - C]. We used 0.1 and 0.001 μmol/l epidermal growth factor (EGF) step pulses as illustrated in Supplementary Fig. 1A and C, respectively, as an input, and phosphorylated MAPK species as output. We performed parameter optimization in MATLAB^®^ as described in the Parameter Estimation section (see main text) on 29 parameters that represent reactions involved in MAPK phosphorylation. Simulated output curves in MATLAB^®^ and COPASI simulations with both original (blue lines) and estimated parameters (red lines) as well as the data points used in the model building are plotted in Supplementary Fig. 1B and D. Simulations in BioNetGen needed modifications that included transformation of inositol triphosphate (IP3) producing reactions to mass kinetic form and adding reactions for active protein kinase C (PKC) production instead of the corresponding SBtab expression. The conversion from SBtab to SBML and from SBML to BNGL models was done by our conversion tools (see paragraph titled Simulations in STEPS). The web-based subcellular simulation setup application was used for importing of the BNGL model to STEPS and running simulations. Note that both stochastic solvers we used produce qualitatively similar solutions which do not converge to the deterministic solutions for the same model. This result is expected for a general type nonlinear and highly stochastic model. In the simulations in NEURON the output of the cascade has been coupled to affect the conductance of Kv4.2 channels in the dendrites, as if a global EGF signal had arrived to the whole neuron. MAPK can phosphorylate Kv4.2 channels which decreases their conductance, and this effect is implemented by scaling the maximal channel conductance. Phosphorylation of the Kv4.2 channels in the dendrites makes the neuron more excitable, which starts firing due to random synaptic input distributed across the dendrites (Supplementary Fig. 4C). Good agreement was found between simulations performed in MATLAB^®^ (Supplementary Fig. 1), COPASI (Supplementary Fig. 1), STEPS, BioNetGen (Supplementary Fig. 3) and NEURON (Supplementary Fig. 4). We also performed a global sensitivity analysis (see Global Sensitivity Analysis in the main text) on the estimated parameters to see what effect different parameters have on the output, either by themselves or in interaction with other parameters (Supplementary Fig. 2).

Finally, we rebuilt a model of the EGF-dependent protein kinase B (Akt) pathway in SBtab (Fujita et al. 2010, provided by Hass et al. 2019 as one of the benchmark problems for modeling intracellular processes), with 11 species, and performed a global sensitivity analysis on the parameters and the figures are available in the Subcellular Workflow GitHub repository^^[[2]](#footnote-2)^^.


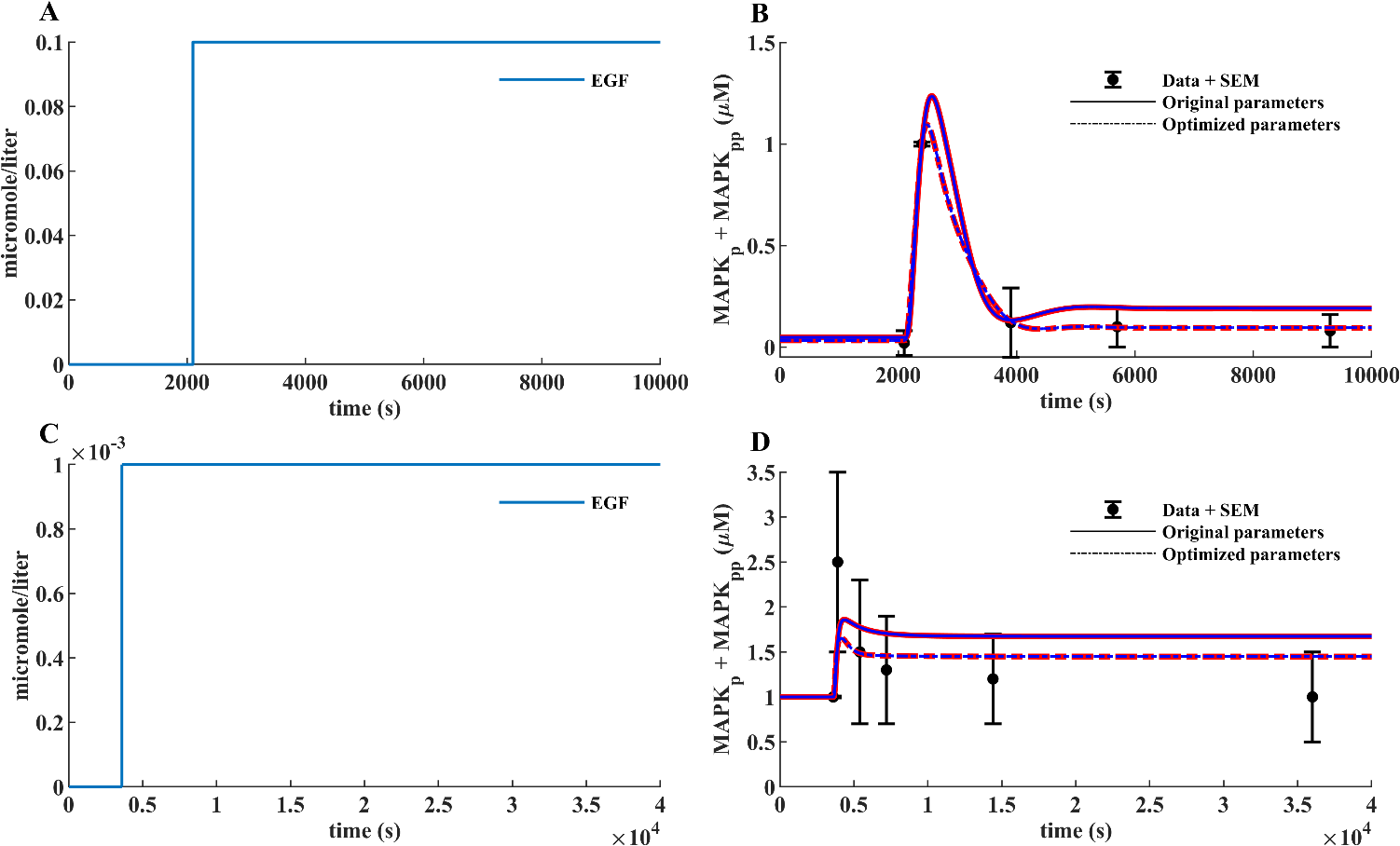


Supplementary Fig. 1 MATLAB^®^ (red lines) and Copasi (blue lines) simulations of the MAPK cascade from the example model provided by FindSim (Viswan et al. 2018). (A, B) and (C, D) correspond to figures 7b and 7c in their manuscript, and to experiments E0 and E1 in our files. (A) and (C) show inputs for the two experiments. Step input of EGF at 0.1 and 0.001 μmol/l is used. (B) and (D) show measured output consisting of the sum of phosphorylated MAPK species. The black dots and error bars refer to data points and standard error obtained from the publication; the continuous lines represent model behavior when simulating with the original parameters; the dashed lines represent model behavior when simulating with parameters obtained by optimization using our MATLAB^®^ parameter estimation tools. The plots are analogous to simulations in Fig. 3 and Fig. 5. All simulations in MATLAB^®^ and Copasi take less than one second to compute (intel core i9-10980XE).


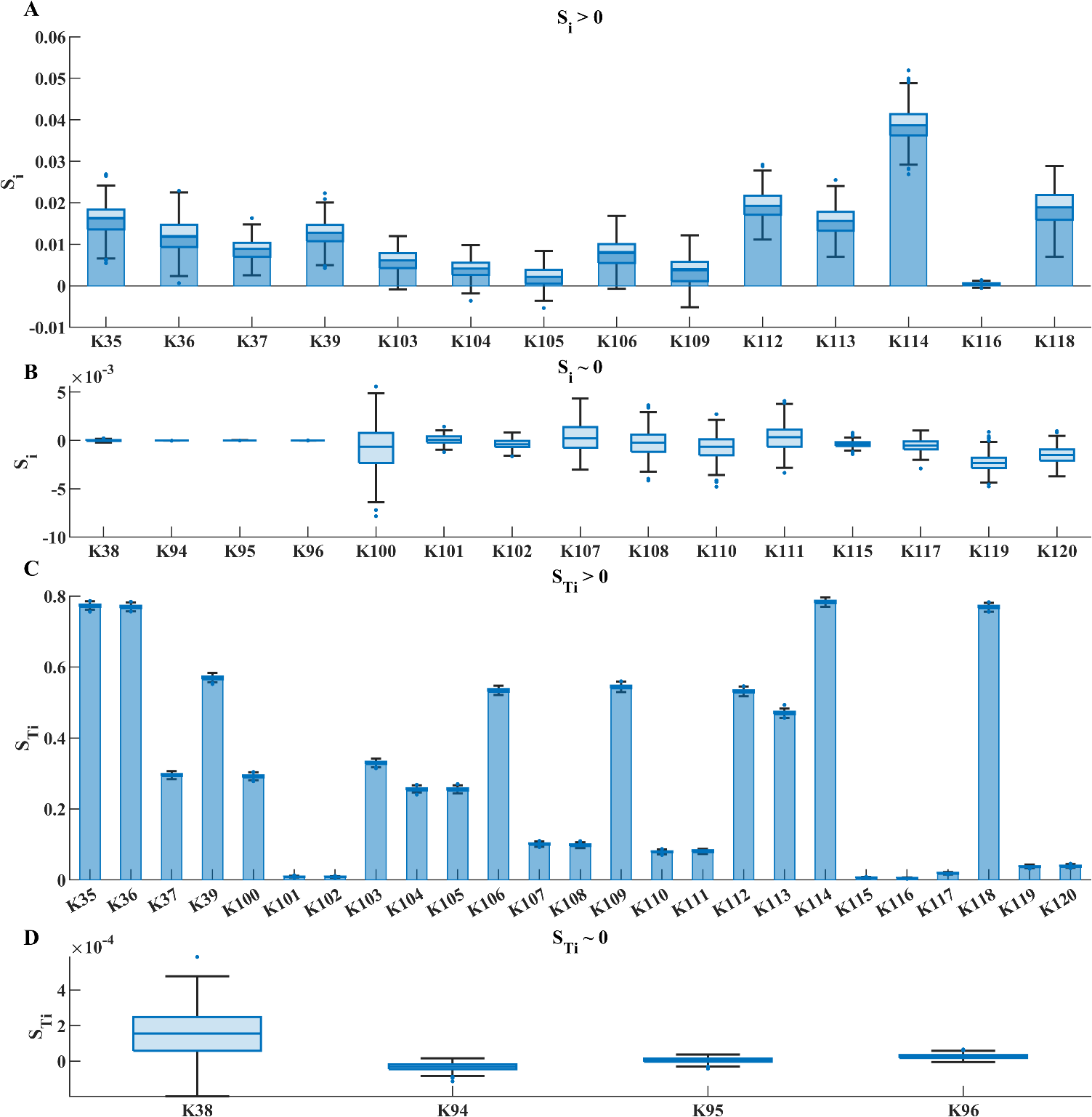


Supplementary Fig. 2: Bar graphs of the first order, S_i_, and total order, S_Ti_, sensitivities indices of the 29 parameters that represent reactions involved in MAPK phosphorylation for one of the experiments (E0) are shown (panel A-B and C-D, respectively). The sensitivities indices are defined in the main text and were calculated based on the scores used in the optimization for the experiment. The parameters, Θ, were sampled independently from a multivariate lognormal distribution with log10(Θ)~ N(μ, σ), using μ =log10(Θ*) and σ=0.1, where Θ* correspond to the optimal values received from the optimization. A sample size of N=100000 was used (corresponding to 3100000 reshuffled samples used in the calculations (Saltelli 2004)). The analysis took approximately 8 hours on 18 compute cores (Intel 10980K). The uncertainty in the sensitivity indices due to sampling was estimated through bootstrapping and indicated by boxplots, showing the median, 25- and 75-percentile, the distance between the lower and upper quartile is the interquartile range (IQR), values that are more than 1.5 times the IQR distant from the top or bottom of the box are considered outliers and all other values are included in the whiskers of the boxplot. This analysis took 4 hours and 3 minutes in MATLAB^®^ using an intel core i9-10980XE.


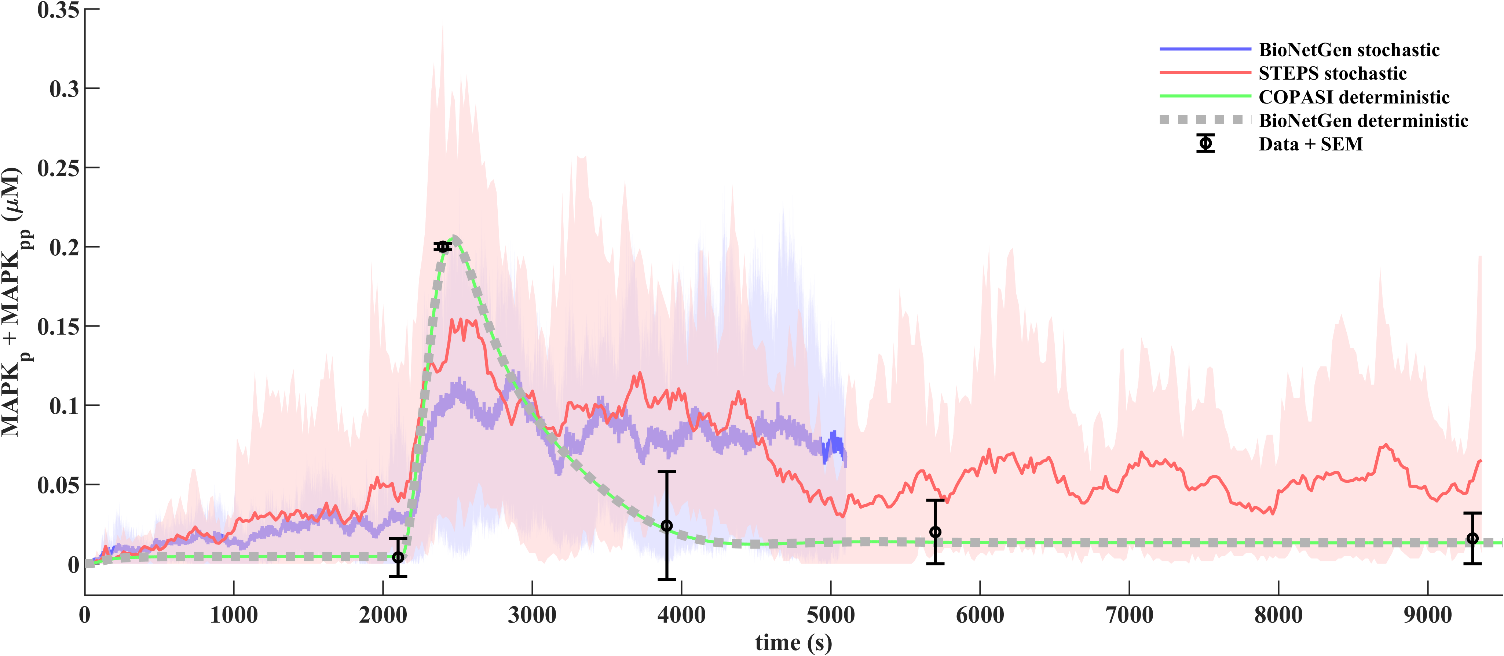


Supplementary Fig. 3: STEPS and BioNetGen simulations of the MAPK cascade from the example model provided by FindSim (Viswan et al. 2018). The optimized parameter set obtained using our MATLAB^®^ parameter estimation tools was used for the simulations. Data and simulations correspond to figure 7b in Viswan et al. 2018, to experiment E0 in our files and to red lines on Supplementary Fig 1 and 3. Step input of EGF at 0.1 μmol/l was applied starting from 2100 second of simulation. Simulated traces show measured output consisting of the sum of phosphorylated MAPK species. The black dots and error bars refer to data points and standard error obtained from the publication; the dotted green line represent model behavior when simulating with COPASI deterministic LSODA solver; the magenta line was obtained by deterministic simulation of BioNetGen solver obtained by the automatic conversion of the example SBtab model modified to make it compatible with BioNetGen language. Note the close correspondence of optimized example model and modified BNGL model solutions; the blue curve corresponds to BioNetGen stochastic ssa solver simulation of the modified BNGL model averaged by 15 simulated traces (5000 seconds of simulations are shown). The blue colored area represents 10%-90% confidence interval of the solution; the red line represents STEPS stochastic solver simulation of the modified BNGL model averaged by 35 simulated traces. The red colored area represents 10%-90% confidence interval of the STEPS solution.


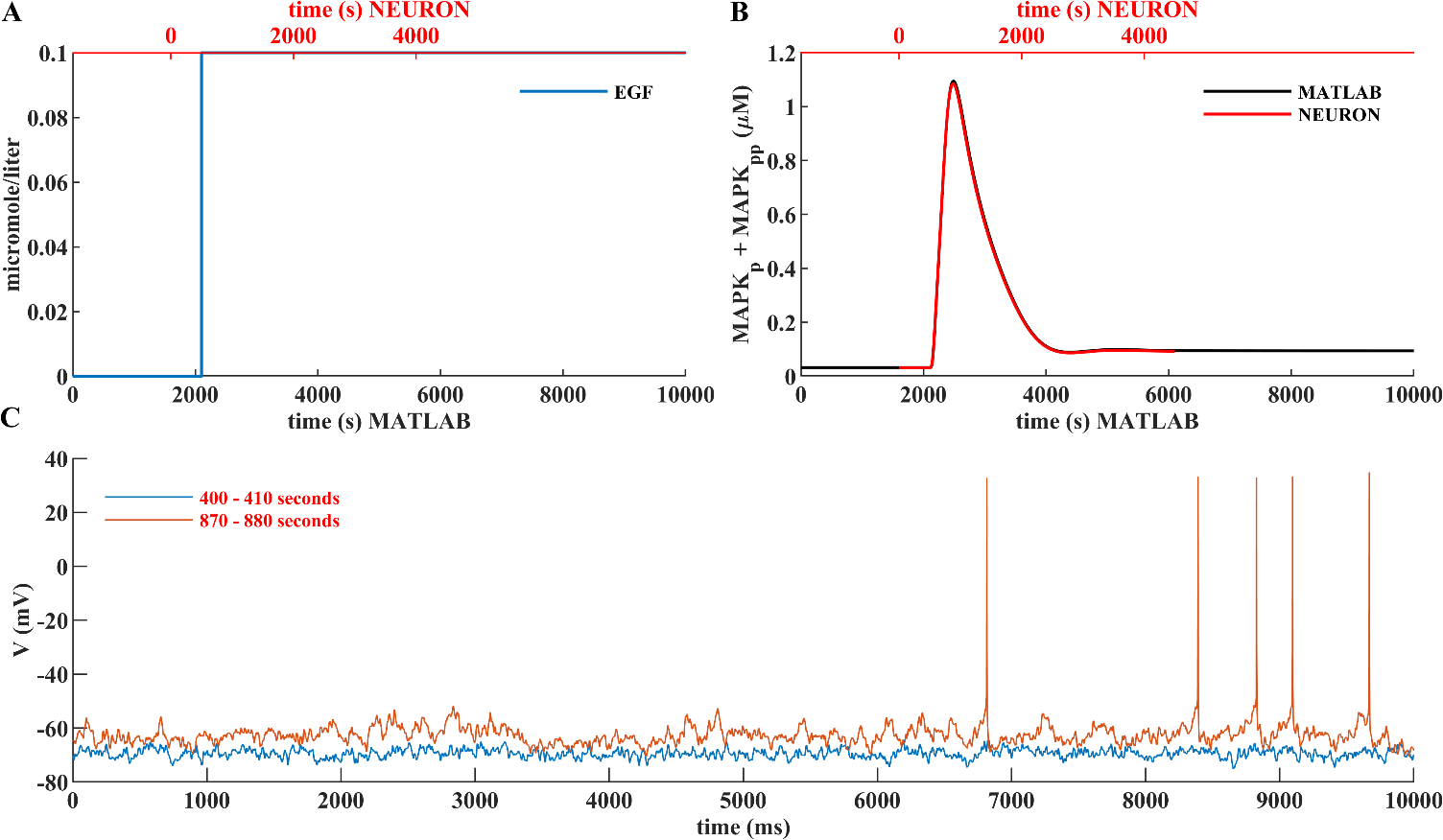


Supplementary Fig. 4: NEURON simulation of the MAPK cascade. The output of the cascade has been coupled to affect the conductance of Kv4.2 channels in the dendrites, as if a global EGF signal had arrived to the whole neuron. MAPK can phosphorylate Kv4.2 channels which decreases their conductance, and this effect is implemented in by scaling the maximal channel conductance according to g = (1 - ([MAPKP] + [MAPKPP])/[MAPK]total) gmax. (A) The simulation lasts 4500 s, and the EGF input arrives at 500 s in Neuron and 10000 s with input at 2100 s in MATLAB (B) Comparison of the normalized output of the cascade in NEURON and MATLAB^®^. (Runtime in Neuron is approximately 20 hours on an Intel core i7-4700MQ CPU @ 2.40GHz and less than one second in MATLAB^®^ using an intel core i9-10980XE) (C) 10s-long somatic voltage traces before (at 400 s) and after (at 840 s) the EGF stimulus show the effect of the cascade on somatic voltage.

1. <https://github.com/jpgsantos/Model_Viswan_2018>/tree/1.0 [↑](#footnote-ref-1)
2. The model GitHub can be found in <https://github.com/jpgsantos/Model_Fujita_2010>/tree/1.0 and example plots of the GSA are in <https://github.com/jpgsantos/Model_Fujita_2010/tree/1.0/Matlab/Results/Examples> [↑](#footnote-ref-2)
